# Nanoimprinted DMD Electrodes Enabling Bidirectional Viewing OLEDs With Quasi Lambertian Emission

**DOI:** 10.1002/advs.202518921

**Published:** 2026-03-02

**Authors:** Ningning Song, Ningning Liang, Xinghao Guo, Ruixiang Chen, Xia Xin, Yiming Chen, Ruiqi Tian, Tianrui Zhai

**Affiliations:** ^1^ School of Physics and Optoelectronic Engineering Beijing University of Technology Beijing China

**Keywords:** bidirectional displays, DMD electrode, Lambertian emission, nano‐imprint technology

## Abstract

Bidirectional displays, capable of simultaneous front and rear illumination, enable transformative applications such as see‐through retail displays, intelligent signage, and next‐generation foldable devices. Despite their potential, the inherent trade‐off between transparency and brightness, coupled with imbalanced bidirectional emission, has hindered the advancement of bidirectional viewing organic light‐emitting diodes (BV‐OLEDs). Here, we present a dual‐approach strategy to achieve simultaneously improved and balanced bidirectional emission with quasi‐Lambertian distribution in bidirectional emitting OLEDs. By combining a microcavity resonance enhancement through nano‐patterned structures and a dielectric/metal/dielectric (DMD) capping layer for improved top electrode transmittance, we effectively suppress surface plasmon and waveguide modes in OLED devices. Consequently, the optimized nanoimprinted DMD electrode achieved a remarkable 89.7% enhancement in transmittance (from 33.9% to 64.3% at 664 nm) compared to conventional planar electrodes; and the resulting BV‐OLED demonstrated balanced bidirectional emission from 33% to 42% with a 67.3% total brightness increase, while exhibiting enhanced transparency with nearly zero haze, quasi‐Lambertian radiation pattern, and excellent color stability across a 120° viewing angle. This breakthrough establishes a fundamental design framework for bidirectional displays, bridging conventional dual‐panel technologies with emerging applications in next‐generation transparent and flexible display systems.

## Graphical abstract

We present a dual‐approach strategy for high‐performance bidirectional‐viewing OLEDs, combining enhanced cavity resonance with a nanoimprinted dielectric/metal/dielectric (DMD) electrode. This bidirectional viewing OLED presents an 89.7% enhanced electrode transmittance, a balanced bidirectional emission ratio with 67.3% total brightness increase, as well as quasi‐Lambertian distribution featuring zero haze and stable color performance within 120° viewing angle.

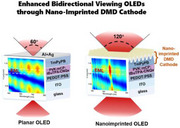

## 1 Introduction

Bidirectional display technology represents a significant advancement over conventional single‐sided displays by simultaneously emitting light from both front and rear surfaces [1, 2, 3]. This technology breaks the traditional limitations of unidirectional displayunique, and thus enabling transformative applications across multiple domains, including see‐through retail displays that maintain visibility while showing dynamic content, intelligent digital signage systems capable of dual‐sided information delivery, and next‐generation foldable devices with seamless 360° viewing experiences [4, 5, 6, 7]. Organic light‐emitting diodes (OLEDs) offer advantages such as low power consumption, wide viewing angles, and wide color gamuts, and have been extensively studied for next‐generation full‐color information displays and large‐area lighting applications [8, 9, 10]. Furthermore, they present a highly promising solution for bidirectional displays, because the design of transparent anodes and cathodes eliminates the need for thin‐film transistors and driving circuits, enabling simultaneous dual‐side emission [11, 12, 13, 14].

However, achieving high‐performance bidirectional viewing OLEDs (BV‐OLEDs) remains a significant challenge, fundamentally constrained by the inherent trade‐off between transparency and luminance that is universal to transparent displays (as evidenced by commercial transparent OLEDs, which typically offer ∼45% transparency with ∼200 cd/m^2^ luminance [3]), coupled with the poor balance between bidirectional sides caused by the inherent structural asymmetry and sever device degradation involved with Joule heating and inefffcient thermal dissipation [15, 16, 17, 18]. Despite the rapid development of various transparent electrode materials in recent years, including conductive polymers, two‐dimensional graphene, MXene, metal nanowires (or nanoparticles), and single‐walled carbon nanotubes [19, 20, 21, 22, 23], several critical challenges persist, particularly for their application as top electrodes in OLEDs. Conventional transparent conductive oxides such as ITO and IZO are constrained by their high processing temperatures, which can degrade underlying organic layers, while solution‐processable electrodes face stability issues due to solvent erosion [24, 25, 26, 27]. Moreover, emerging materials like graphene and MXene still struggle with achieving uniform large‐area fabrication, maintaining low contact resistance, and ensuring long‐term stability [28, 29, 30, 31, 32]. Recently, metal‐dielectric multilayer thin‐film structures (e.g., metal/oxide or metal/wide‐bandgap semiconductor stacks) like dielectric‐metal‐dielectric (DMD) trilayer structures have been proposed as promising transparent electrodes via suppressing the surface plasmon polariton (SPP) coupling at the metal‐dielectric interface, and tuning the mismatch in dielectric function between the dielectric and metallic layers. However, when applied these DMD electrodes to bidirectional viewing displays or transparent LEDs, effectively addressing the uneven dual‐sided emission caused by the inherent structural asymmetry and mitigating the degradation in device stability resulting from Joule heating in the metal electrodes are still key challenges.

Additionally, the inherent structural asymmetry in transparent OLEDs characterized by refractive index mismatches between layers and dissimilar material compositions of the anode/cathode, significant imbalance exists between front and rear light emission intensities. This presents a critical challenge for achieving 360° display applications. Currently, various advanced techniques for extracting confined light from OLEDs have been reported, such as microlens arrays [33, 34], light scattering centers [35, 36], grid [37, 38], and photonic crystals [39, 40]. Whereas the integration compatibility of these methods with BV‐OLED technology requires particular attention, as both high‐temperature deposition and solution‐processing of these optical structures may significantly compromise device efficiency. Of these, Micro/nano patterns with both periodic and random structure is confirmed to be effective to increase light extraction of an OLED. Whereas, an appropriate periodicity such that waveguided light is Bragg‐scattered into the forward direction is required for the periodic micro/nano patterns by satisfying the Bragg scattering condition, and thus gives rise to a non‐Lambertian emission and blue shift of electroluminescent (EL) spectra with varying viewing angles, which is not suitable for high‐quality display. Therefore, a unifying model to solve or alleviate the imbalance in brightness and to maintain a non‐Lambertian emission with excellent color stability within a 120° viewing angle for bidirectional display is still lacking. Accordingly, it is highly desirable to develop a synergistic strategy to design a high‐performance top electrode (transmittance and conductivity) that is compatible with heterogeneous OLEDs to balance and improve the bidirectional emission for BV‐OLEDs.

Herein, we present a dual‐approach strategy for high‐performance bidirectional‐viewing OLEDs, combining enhanced cavity resonance with a nanoimprinted DMD electrode featuring higher transparency, lower sheet resistance, and minimal haze. The synergistic design strategy effectively extracts the localized SPP modes coupling at the metal/organic interface and waveguide modes localized in organic layers, and thereby, enhances the light extraction efficiency of the resultant OLED. In detail, this bidirectional viewing OLED presents an 89.7% enhanced electrode transmittance (from 33.9% of planar OLED to 64.3% at the wavelength of 664 nm), a balanced bidirectional emission ratio (42% *vis* 33%) with 67.3% total brightness increase, as well as quasi‐Lambertian distribution featuring zero haze and stable color performance within 120°. These advancements establish a new paradigm for next‐generation transparent/flexible displays, overcoming traditional transparency‐brightness trade‐offs in BV‐OLED development.

## 2 Results

### 2.1 Design and Construction of Corrugated Top Electrode Compatible with OLED

**FIGURE 1:**
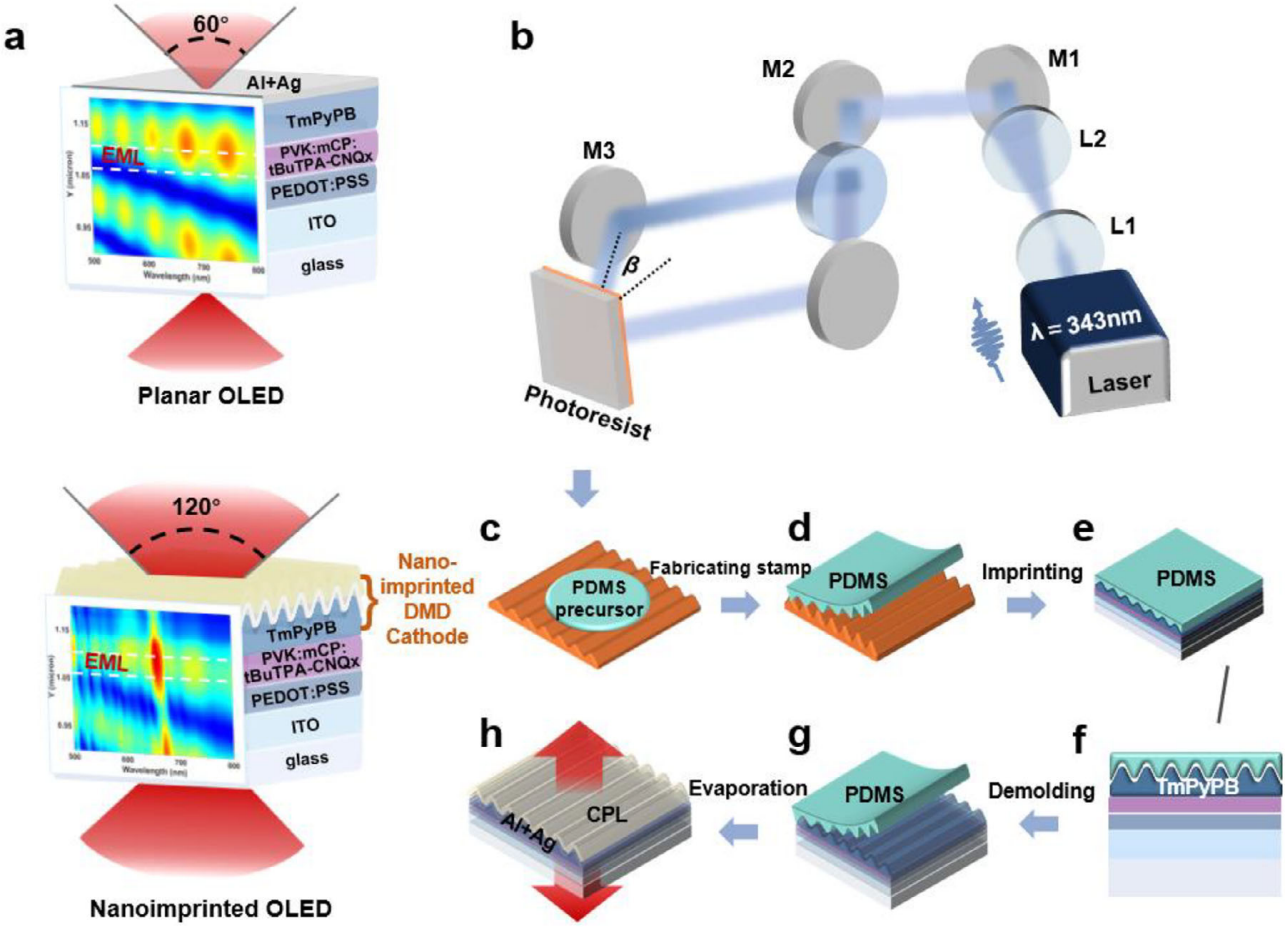
**Design idea and illustration of the fabrication process for nanoimprinted bidirectional emitting OLEDs**. (a) Schematic of conventional planar and nanoimprinted OLED with different emission patterns. (b) Schematic of the tunable‐periodicity grating fabrication process based on dual‐beam laser interferometry. (c) Spin‐coating of PDMS onto the photoresist grating master. (d) Peeling off of the cured PDMS grating layer. (e) Sticking of PDMS onto the electron transport layer by the thermal imprinting setup. (f) Diagram of the nanoimprinted electron transport layer. (g) Demolding procedure of PDMS after nanoimprinting. (h) Thermal evaporation of thin metal film as well as the capping layer for bidirectional emitting OLEDs.

Considering the compatibility of the top‐emissive cathode with heterogeneous OLED, we still adopted the evaporation‐deposited thin silver (Ag) film and utilized a DMD architecture. As shown in Figure 1a, to form an enhanced bidirectional emission with a wider emission angle, more balanced and stronger double‐sided emission, nanoimprinted OLED structures embedded with a nanoimprinted DMD geometry were introduced by a low‐cost and large‐area method combining laser interferometric lithography, thermal nanoimprinting, solution processing, and finite difference time domain (FDTD) simulation. In this study, a solution‐processed deep‐red OLED with a grating structure was fabricated with the architecture of a glass substrate/ ITO/ poly(3,4‐ethylenedioxythiophene) poly(styrene sulfonate) (PEDOT:PSS)/poly(9‐vinylcarbazole) (PVK): N'‐dicarbazoyl‐3,5‐phenylene (mCP): 6,7‐bis(4‐(bis(4‐(tert‐butyl) phenyl) amino)phenyl)‐2,3‐bis(4‐(tert‐butyl)phenyl) quinoxaline‐5,8‐dicarbonitrile (tBuTPA‐CNQx) [41]/(3,3'‐[5'‐[3‐(3‐Pyridinyl)phenyl][1,1':3',1''‐terphenyl]‐3,3''‐diyl]bispyridine) TmPyPB/LiF/Al/Ag (detailed information could be found in Method section). The PEDOT:PSS as the hole injection layer (HIL) and hole transport layer (HTL), TmPyPB as the electron transport layer (ETL), and LiF as the electron injection layer (EIL) to improve hole and electron injection and transport. The emissive layer (EML) is composed of commercial host materials PVK and mCP (in a 1:2 ratio) and a red emissive guest material, tBuTPA‐CNQx (20% wt). While previous studies [42, 43] have extensively explored the use of traditional iridium complex emitting layers in imprinted OLEDs, this work focuses on demonstrating the applicability of nanoimprint lithography to fluorescent materials. Herein, for nanoimprinted OLED, the ETL (TmPyPB) is imprinted by controlling the imprinting conditions, such as pressure, holding time, holding temperature, etc. In contrast, the planar device does not undergo imprinting at this stage. Additionally, as Ag tends to grow as isolated islands due to the Volmer‐Weber growth mode, we adopted an Ag film as thin as 20 nm using a 2‐nm‐Al as a seed layer to enhance the density of nucleation sites and facilitate the growth of continuous thin Ag films with a smooth morphology. Consequently, the additional deposition of the capping layer (CPL) Dipyrazino[2,3‐f:2',3'‐h]quinoxaline‐2,3,6,7,10,11‐hexacarbonitrile (HATCN) forms the DMD architecture to achieve a high‐performance bidirectional emitting OLED.

**FIGURE 2:**
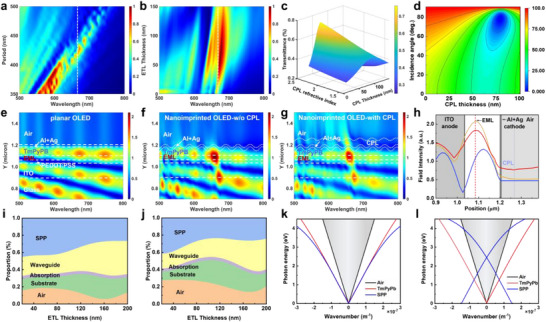
Mechanism analysis and optimization for the planar and nanoimprinted OLEDs. The role of architecture parameters (a) grating period and (b) ETL thickness on the far‐field electric field distribution at different wavelengths. (c) Transmittance of the nanoimprinted OLED as a function of refractive index and thickness at 664 nm wavelength for CPL; (d) TE‐mode Reflection at the upward direction as a function of CPL thickness and incident angle. Internal electric field distribution under planar light incidence for (e) planar OLED, (f) nanoimprinted OLED w/o CPL, and (g) nanoimprinted OLED with CPL. (h) Comparison of the electric field intensity distribution at the wavelength of 644 nm for these three OLEDs. (The blue, orange, and red lines are corresponding to planar OLED, nanoimprinted OLED w/o CPL, and with CPL, respectively). Simulated light mode distribution of (i) planar and (j) nanoimprinted OLEDs. Theoretical dispersion curves of SPP modes for (k) planar OLEDs and (l) nanoimprinted OLEDs. The diagonal lines correspond to the light cones of air (black line) and TmPyPB (red line).

**FIGURE 3:**
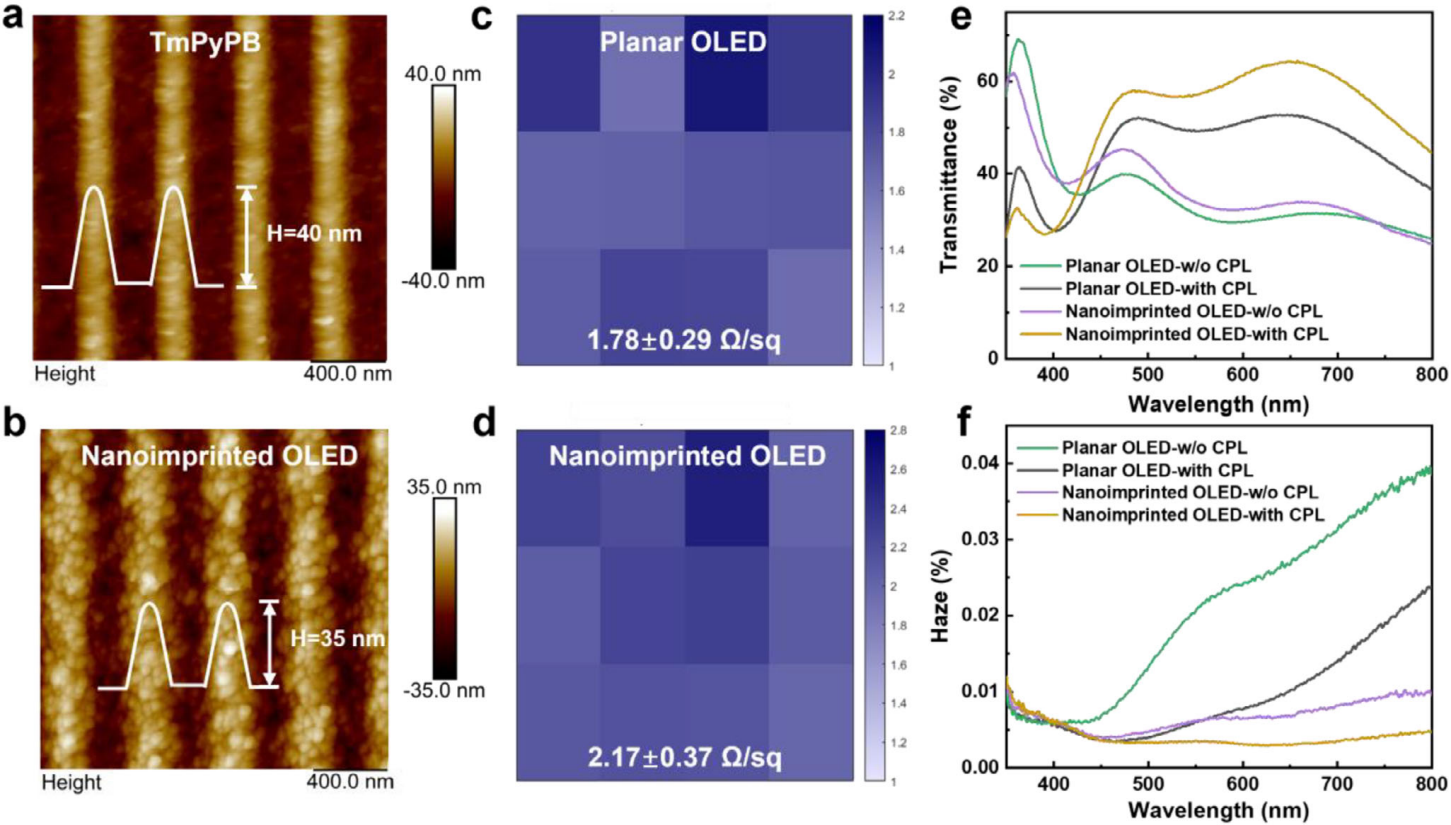
Characteristics of optical and electrical properties for nanoimprinted OLED. (a) Height of atom force microscope for (a) the nanoimprinted TmPyPB layer, and (b) the deposited Al/Ag thin electrode. Sheet resistance of Al/Ag thin electrode in (c) planar OLED and (d) nanoimprinted OLEDs. (e) Transmittance and (f) haze comparison of planar and nanoimprinted OLED devices with or without a CPL layer.

Figure 1b demonstrates the mask‐free laser lithography technique [44, 45] used to create regular interference patterns. By exposing spin‐coated photoresist to two oblique 343 nm laser beams, tunable grating structures were achieved through controlled dual‐beam interference angles (see Note S1 and Figure S1). The OLED fabrication process involves: (1) Spin‐coating and curing Polydimethylsiloxane (PDMS) on a patterned photoresist template (Figure 1b); (2) Transferring the grating structure to a pre‐prepared OLED with an electron transport layer [46] (Figure 1c, d); (3) Imprinting under varying conditions to produce different grating heights [47, 48] (Figure 1e and Figure S2). (4) Cooling and carefully removing the PDMS mold (Figure 1f, g); (5) Depositing remaining components to complete the double‐sided light‐emitting OLED (Figure 1h). During the imprinting process, the temperature is raised to 60–70°C under a pressure of 5 bar. Since no solvent exposure is involved, no additional adverse effects are imposed on device performance. Herein, the device features a 22 nm transparent cathode (2 nm Al/20 nm Ag) and a HATCN cover layer to enhance optical transmittance and protect underlying layers. This optimized structure ensures both conductivity and light emission efficiency. Meanwhile, via precisely controlling the lithography condition (recording angle *β*, laser power, exposure time, development time of the photoresist film), and the nanoimprinting condition (pressure, pressing time, and pressing temperature), a perfect photoresist grating with a 430 nm period and a certain depth (140 nm) and thus high‐performance bidirectional deep‐red OLED devices are realized, as shown in Figure 1a. This novel method, combining laser interference lithography and thermal nanoimprint lithography, and solution‐processed emitting layer, is extremely flexible, cost‐effective, and highly efficient for the construction of corrugated OLEDs.

### 2.2 Optimization of Nanoimprinted DMD Electrode for Cavity Resonance Enhancement

Owing to the top metal electrode could induce more SPP mode, a severer photon loss and thus a suppressed outcoupling efficiency are generally induced in the top emission direction. Along with the relatively higher refractive index for ETL (TmPyPB) than that of EML and HTL (PEDOT:PSS), as summarized in Figure S3, a corrugated DMD electrode is introduced, as shown in Figure 1a. Accordingly, the grating period at a specific emission wavelength (*λ*_0_ = 664 nm), the thickness of ETL under different planar light incidences, as well as the dielectric type and thickness were simulated to achieve a maximum light extraction efficiency for the resultant corrugated OLEDs. Herein, considering the optimal nanoimprinting conditions of the nanoimprinted ETL during the experiment (Figure S2), such as the integrity and uniformity of the imprinted structure and the rectangular shape of the grating lines, the ETL grating height is determined to be 40 nm, which is included into the total ETL thickness. As presented in Figure 2a and S4a, b, being consistent with the general formula of the grating equation, the strongest emission wavelength is significantly redshifted with the increased grating period; whereas, it presents a slight red‐shift as the thickness of ETL increases. As a result, for the wavelength of 664 nm, the optimized parameters were determined to be 430 nm for grating period and 80 nm total thickness for ETL layer (Figure 2a, b).

Another task of this work is to optimize the material type and its thickness for the capping layer, aiming to significantly elevate the outcoupling efficiency of nanoimprinted OLEDs and further protect the underlying thin‐metal film. The optimal CPL material and thickness are determined by calculating the transmittance and reflectance of multi‐layer dielectric films using the transmission matrix method, with the specific calculation method detailed in Note S2. As shown in the simulation results (Figure 2c and Figures S5, S6), higher refractive indices reduce reflectance and enhance anti‐reflection performance, requiring an optimal CPL thickness. These results indicate that 60 nm HATCN provides a relatively higher anti‐reflection effect at the emission region. Accordingly, variable angle spectroscopic ellipsometry [49] was further utilized to confirm the minimization of total reflection of light and maximization of the transmittance for the DMD electrode at the condition of 60‐nm HATCN as CPL. As shown in Figure 2d, at the emission wavelength of OLED, TE‐mode reflectivity exhibits minor and stable variations with DMD dielectric thickness and incident angle. And TM‐mode shows similar behavior (Figure S7). However, TE‐mode demonstrates more significant reflection suppression, while partial loss occurs in TM‐polarized light due to backward reflection forming waveguide modes. Considering the effective refractive index (*n*) of 1.81 for HATCN material, coupled light escapes from the device surface at approximately 60° (corresponding to an escape cone of 120° and an internal angle < 33°). A dielectric layer thickness of 60 nm effectively optimizes the minimization of reflections for both TE‐ and TM‐polarized light. In addition to the grating period and DMD thickness, we also investigated the influence of grating height on the outcoupling efficiency (Figure S8). Although increasing the grating depth can moderately adjust the near‐field distribution, the overall light‐extraction efficiency decreases when the grating height exceeds a critical value (∼40 nm in this configuration). This indicates that further increasing the grating height yields diminishing returns in overall outcoupling and may even compromise the electrical property of the thin Ag/Al electrode. Therefore, to balance optical enhancement with electrical reliability, the grating height was fixed at 40 nm.

### 2.3 Mechanism Analysis of Nanoimprinted DMD Architecture for Light Output Enhancement

In order to investigate the role of nanoimprinted DMD electrode on the outcoupling efficiency, the corresponding internal electric field distributions for the planar, nanoimprinted OLED with and without CPL were simulated. Comparative studies demonstrate that the grating structure effectively modulates the internal electric field distribution, of which a uniform internal electric field distribution is presented in the planar OLED (Figure 2e), while the nanoimprinted OLED shows a significant difference in both internal and external electric field, with a significantly suppressed mode distributed in the glass substrate (Figure 2f, g and Figure S9). In detail, the nanoimprinted OLED displays a more pronounced electric field location as well as a stronger far‐field intensity for the specific wavelength of 664 nm, especially for the transverse magnetic (TM) mode, as shown in Figure S9d. More interestingly, compared to the planar OLED, the introduction of corrugated electrodes with or without CPL (Figure 2e–g) presents a more localized electric field to the EML with a more pronounced external light output. As expected, as contrast in Figure S10, the nanoimprinted DMD electrodes effectively extract the TM mode distributed at the top metal/TmPyPB interface, demonstrating the suppression of SPP mode. Figure 2h further illustrates that the nanoimprinted DMD electrodes facilitate the location of electric field shift to the EML, significantly enhance the electric field strength, and simultaneously enhance the bidirectional emission, confirming the powerful optical modulation of our designed corrugated DMD electrode.

Simulations at 664 nm wavelength analyzed the intensity variations of different optical modes, including air, substrate, absorption, SPP, and waveguide (WG) mode with increasing ETL thickness in both planar and imprinted OLEDs (Figure 2i,j). Following the introduction of the corrugated DMD structure, air mode emission at the target wavelength was significantly enhanced. Conversely, waveguide and plasmonic modes weakened, indicating that the nanoimprinted architecture couples trapped light into output (Figure S11). At ETL = 40 nm, the light extraction efficiency of the 664 nm air mode showed substantial enhancement. The nanoimprinted TmPyPB/Ag/HATCN addresses energy loss from momentum mismatch in planar structures by providing additional momentum (*k*_g_, *k*_g_ = 2π/period) through Bragg scattering (Figure S12). As expressed by the Bragg equation (*k*_TmPyPB_ = *k* ± *k*_g_), this grating‐imparted momentum shifts the dispersion curve of Ag surface plasmon polaritons, as shown in Figure 2k, l, causing it to overlap with the TmPyPB light cone. This satisfies the momentum‐matching condition, decoupling trapped modes and enhancing device light extraction efficiency [50, 51]. Thus, with the optimized DMD structure and the incorporation of the grating corrugation, the resulting nanoimprinted OLEDs are expected to achieve quasi‐Lambertian emission with excellent color stability within a 120° viewing angle. This broadening of the emission profile is consistent with the established principle that efficient extraction of confined WG and SPP modes via nanostructuring leads to a reduction in far‐field directionality and a more Lambertian radiation pattern [52].

### 2.4 Characteristics of Optical and Electrical Properties for Nanoimprinted OLED

After establishing the structure‐performance relationships linking device architecture to optical field distribution and overall device efficacy, we further characterized the key metrics of nanoimprinted OLEDs for bidirectional displays, including surface morphology, sheet resistance, transmittance, and haze. According to the surface morphology of the imprinted TmPyPB layer and the resulting top Ag films, outstanding performance, including the grating height, period, and surface roughness (Figure 3a, b) has been realized for the resultant nanoimprinted OLEDs. Meanwhile, compared to the planar OLEDs, nanoimprinted OLEDs also display a uniform and relatively small sheet resistance as low as ∼2.5 Ω/sq (Figure 3c, d), indicating a lower leakage current and elimination of short circuit for the subsequent OLED devices. Herein, the nanoimprinting is performed only on the TmPyPB layer at a controlled temperature (60–70°C) and pressure (5 bar), which is below its glass transition temperature and does not affect the underlying emissive layer, as seen in Figure S13.

As a bidirectional display, high transparency and low haze are also as the important technical indicators, and hence the transmission, reflection, and haze of the resultant OLED devices were characterized, as shown in Figure 3e, f and Figure S14. Of these, the haze formula is calculated as follows [53]: Haze = $\frac{T_{diffuse}}{T_{total}}$×100. Detailed information could be found in Note S3. The transmittance of the nanoimprinted OLED shows a slight increase with decreased scattering and haze, compared with planar counterparts. Upon adding CPL, the overall transmittance of the device significantly increases with a further suppression of haze. Consequently, the optical transmittance of the nanoimprinted DMD modulated OLEDs at the target wavelength of 664 nm rose from 33.9% for conventional planar OLEDs to 64.3%, attributed to the superior anti‐reflective property and light extraction efficiency for the corrugated TmPyPB/Ag/HATCN architecture, particularly for the TM‐mode. Further validation demonstrates that establishing anti‐reflective conditions can effectively enhance the light extraction efficiency of the corresponding OLEDs, serving as a universal method for achieving efficient light extraction. Based on the above results, it is reasonable to conclude that the low‐cost thermal nanoimprinting technology integrated with flexible laser interferometric lithography demonstrates high compatibility for heterogeneous OLEDs, enabling precise optical field distribution tuning.

### 2.5 Electroluminescence Performance for the Designed Bidirectional Viewing Organic Light‐Emitting Diodes

**FIGURE 4:**
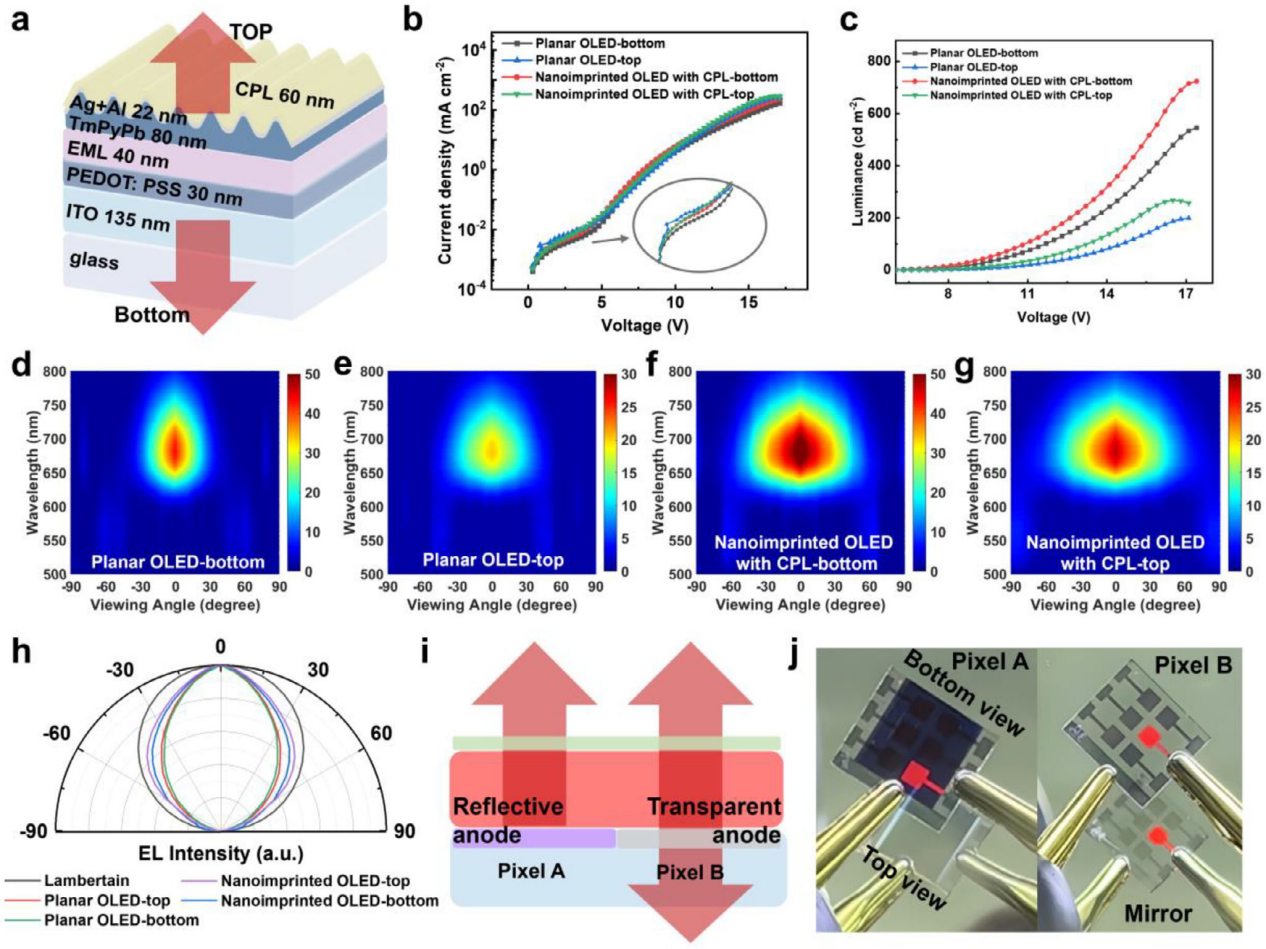
Electroluminescence performance of nanoimprinted OLED for bidirectional viewing application. (a) Schematic diagram of the device structure for dual‐sided display OLEDs. (b) Current density, (c) Luminance of planar and nanoimprinted OLED devices at different voltages. Angle‐resolved electroluminescence emission intensity of planar OLED devices at all angles and wavelengths (d) bottom emission and (e) top emission, and angle‐resolved electroluminescence emission intensity of nanoimprinted OLED devices at all angles and wavelengths (f) bottom emission and (g) top emission. (h) Angular distribution of electroluminescence emission intensity at 664 nm for each OLED. (i) Schematic diagram of the principle of integrated bidirectional display pixels and (j) single‐sided emission pixel A and double‐sided emission pixel B. A mirror is placed behind the device to observe the rear view.

We have finally fabricated a bidirectional emitting OLED, whose architecture as well as the thickness of each layer are shown in Figure 4a, while further details are illustrated in the Method Section. The electroluminescence performance was evaluated stepwise. First, after introducing only the grating structure through embossing (see Figure S15), the device brightness was enhanced considerably, with the bottom emission brightness increasing from 545.6 to 698.9 cd·m^−^^2^, and the top emission brightness increasing from 198.9 to 216.1 cd·m^−^^2^, respectively; a reduction in leakage current was observed for both emission directions. Subsequently, by further introducing the corrugated DMD transparent electrodes to form the complete optimized structure, the devices presented the characteristics shown in Figure 4b, c. While the final DMD structure resulted in an asymmetric modification of the sub‐threshold leakage current (with a reduction in the top‐emission direction and a marginal increase in the bottom‐emission direction, as shown in the inset of Figure 4b), it dramatically enhanced the light outcoupling at operating voltages. The maximum emission intensities were further enhanced with the bottom and top emission increased to 723.9 and 267.7 cd·m^−^^2^, respectively, representing improvements of 32.7% and 34.6%, respectively, when compared to the conventional counterparts. The histogram distribution of luminance and its variance for the statistical results of several devices are shown in Figure S16. Meanwhile, the bottom and top emission intensity ratio for the nanoimprinted OLED with CPL was enhanced from 33% to 42% at the injection voltage of 14 V for conventional planar ones, with a 67.3% total brightness increase. This is attributed to the conversion from the SPP and waveguide modes to the air mode as well as the Purcell effect induced by the enhanced cavity resonance, as illustrated in Figure 2. Moreover, this luminance imbalance originates from differences in optical transmittance and reflectance between the ITO anode and DMD cathode, coupled with synergistic multi‐reflection and interference effects within the optical cavity formed by heterogeneous mirrors. Consequently, advanced transparent electrodes must be engineered through rigorous optical design and precise deposition techniques to eliminate structural asymmetry‐induced luminance imbalance.

Meanwhile, the emission spectra of OLEDs exhibit similar profiles before and after nanoimprinting, whereas a slight reduction in full width at half maximum (FWHM) is observed in nanoimprinted devices. This spectral narrowing effect is attributed to light diffraction by the nano‐grating structures. Figure 4d–g illustrates the angle‐resolved electroluminescence emission intensity of these four types of OLED, to investigate the effect of nanoimprinted DMD architecture on the emission profile and the viewing angle. Of these, the planar OLED has a divergence angle of 60°, presenting a relatively severe narrow viewing angle characteristic, as shown in Figure 4h. Critically, the nanoimprinted OLED with CPL achieves a wide viewing angle of approximately 120° and exhibits remarkable color stability, with a CIE coordinate shift of less than (0.01, 0.01) over the 0°–60° range (see Figure S17). This outstanding performance arises from a synergistic optical management strategy: the nanograting provides additional in‐plane momentum to efficiently couple waveguide and surface plasmon polariton modes into the radiation mode, thereby broadening the angular distribution of emitted light. In synergy, the DMD structure helps manage optical modes and suppress parasitic losses. Together, they effectively tailor the microcavity characteristics and mitigate the mechanisms behind angle‐dependent spectral shifts, yielding a quasi‐Lambertian radiation profile. These experimental results closely align with our previous theoretical analysis, confirming that the nanoimprinted DMD architecture achieves both high brightness and excellent angular color stability, demonstrating its strong potential for high‐quality bidirectional displays. Importantly, this significant enhancement in optical performance is achieved without compromising electrical and operational integrity. Ultraviolet photoelectron spectroscopy (UPS) confirms that the nanoimprinting process induces negligible change (< 0.2 eV) in the work function alignment at the critical TmPyPB/cathode interface (as Figure S18), ensuring nearly unaltered charge injection behavior. The operational stability of unencapsulated devices was further evaluated under constant current driving at 5 mA in a nitrogen atmosphere. As shown in Figure S19, the planar OLED retains only 50% of its initial luminescence on both the top and bottom sides after 40 h. In contrast, the nanoimprinted OLED with CPL demonstrates significantly higher stability than its planar counterpart, maintaining over 60% of its initial brightness on both sides under the same driving conditions. This enhancement in stability can be attributed to the synergistic effects of the designed corrugated DMD electrode. Specifically, the HATCN capping layer serves as an effective barrier against environmental degradation, while the nanoimprinted grating structure improves thermal management by suppressing the SPP effect and thus the Joule heating near the metal electrode, thereby promoting more efficient heat dissipation [54]. These results notably confirm the efficiency of our dual design strategy by combining a microcavity resonance enhancement through nano‐patterned structures and a DMD via a capping layer, which can significantly improve the bidirectional emitting characteristics, including transmittance, haze, bidirectional emission intensity, and corresponding balance, as well as the viewing angle.

Additionally, we further explored the potential applications of bidirectional emitting OLEDs in bidirectional displays. Conventional bidirectional emitting OLEDs use top‐emitting structures with non‐emitting transparent regions, where the reflective layer sits above the drive circuit. This creates a conflict between emitting and transparent areas on the reflective layer, and traditional TrOLEDs only display on one side. We utilized a bidirectional OLED display (Figure 4i) by integrating two subpixel types, namely A (single‐sided emission) and B (bidirectional emission) [11]. This enables different information display on each side while maintaining overall transparency. Subpixel A achieves one‐sided emission using another reflective DMD structure that reflects the deep‐red photon back to the cathode direction (Detailed information could be found in the Method Section). The mirror measurements in Figure 4j confirm that when A is activated, the image is only visible from the front; when B is activated, both sides emit clear red light. By selectively activating A and B subpixels within the same transparent device, two distinct patterns can be displayed simultaneously, providing information in diverse settings without sacrificing transparency.

## 3 Conclusion

In summary, we present a dual‐approach strategy achieving simultaneously enhanced and balanced bidirectional emission with quasi‐Lambertian distribution. This work establishes that the synergistic combination of microcavity resonance enhancement via nanoimprinted structures and a DMD capping layer is a fundamentally effective and reliable design framework for next‐generation transparent displays. The optimized nanoimprinted OLED achieved a transmittance of 64.3% at 664 nm with an ∼90% enhancement over conventional planar counterparts of 33.9%. The resulting BV‐OLED demonstrated a balanced bidirectional emission, improving the top/bottom luminance ratio from 33% to 42% with a 67.3% total brightness increase. It also exhibited near‐zero haze, quasi‐Lambertian radiation, and excellent color stability across a 120° viewing angle. The demonstrated device performance, coupled with robust interfacial integrity and operational stability, underscores the strong application potential of this architecture in overcoming the long‐standing transparency‐brightness‐balance trilemma for see‐through displays, intelligent signage, and future flexible/bendable electronic devices.

## 4 Methods

### 4.1 Materials

Unless otherwise specified, solvents and chemical reagents were purchased from commercial sources and were not further purified before use. Chlorobenzene (CB) was purchased from J&K Scientific. PEDOT:PSS (4083), PVK, mCP, tBuTPA‐CNQx, TmPyPB, LiF, HATCN, and Ag and Al particles were purchased from Xi'an Polymer Optoelectronics Technology Co., Ltd.

### 4.2 Preparation of Photoresist Nanogrids on Glass Substrates

Glass substrates were sequentially cleaned with deionized water, acetone, and ethanol. Before spin‐coating the photoresist layer, the substrates were dried and exposed to UV‐ozone for 20 min. After the glass cooled to room temperature, a 145‐nm‐thick ultra‐thin photoresist layer (AP‐R3170) was spin‐coated at 2,800 rpm for 50 s, followed by baking at 100°C for 55 s. A laser source with a wavelength of 343 nm is split into two optical paths, which are ultimately merged and interfere with the photoresist, resulting in a diffraction grating pattern with a period of 430 nm after 9 s of development.

### 4.3 OLED Preparation

The device structure consists of indium tin oxide (ITO, 135 nm)/poly(3,4‐ethylenedioxythiophene) poly(styrene sulfonate) (PEDOT:PSS, 30 nm)/EML (PVK:mCP: tBuTPA‐CNQx, 40 nm)/(1,3,5‐Tris(3‐pyridyl‐3‐phenyl)benzene (TmPyPB, 80 nm)/lithium fluoride (LiF, 0.5 nm)/aluminum (Al, 2 nm)/silver (Ag, 20 nm)/ 1,4,5,8,9,11‐Hexaazatriphenylenehexacarbonitrile (HATCN, 60 nm), where PEDOT:PSS serves as the hole injection layer and hole transport layer, LiF and TmPyPB act as the electron injection layer and electron transport layer, respectively, and HATCN functions as the external anti‐reflective layer of the device. The nanoimprint device is imprinted after TmPyPB is evaporated, and the remaining layers are further evaporated after cooling. During evaporation, the evaporation rate of organic materials is 0.1 Å s^−1^, and that of metal materials is 1–2 Å s^−1^. All materials are evaporated at a pressure of 2·10^−4^ Pa.

### 4.4 Characterization of OLEDs

The EQE‐brightness‐voltage curves and EL spectra of planar and imprint OLEDs were measured using a programmable source meter (Keithley 2400) and a spectrophotometer (Spectrascan PR650). The spectrophotometer measures the power per steradian per unit area at each wavelength (units: W nm^−^^1^·sr^−^^1^·m^−^^2^). Film thickness was measured using an XP‐2 stylus profilometer. Atomic force microscopy (AFM) was performed using the Dimension Icon AFM from Bruker Corporation. Sheet resistance measurements were performed with a four‐point probe system (Wuhan Precise Instrument Co., Ltd, S100).

### 4.5 Theoretical Simulation

The optical transmittance of the electrodes, far‐field intensity distribution, and OLED optical mode distribution were calculated using the FDTD method combined with custom‐developed code. In the simulations, the refractive index (n, k) was measured using an ellipsometer over the wavelength range of 400–850 nm, and material models were automatically generated for FDTD simulations. Due to the periodicity of the nanograting, periodic boundary conditions were applied in the *X* direction and perfect matching layer (PML) boundary conditions in the *Y* direction when simulating the far‐field distribution and internal electric field distribution of the device to optimize the grating structure. When analyzing the optical mode distribution within the device, the *X*‐direction boundary conditions were set to PML, and the *X*‐direction span of the device was set to 25 µm to minimize the impact of size on the simulation. Plane waves are used to simulate transmission, reflection, and the internal electric field distribution of the device, while the remaining light sources are configured as dipoles. To account for the spatial distribution of emitters, dipoles were placed at various locations within the emissive layer, and the final results were obtained from an average of these simulations to ensure statistical representativeness. The transmission box module is used in the optical power section to monitor the emitted power. Fine adjustments to parameters such as film thickness and grating period are achieved through scanning functionality, and corresponding computational results are obtained using a power monitor. For modal dispersion and transmission matrix calculations in reciprocal space, MATLAB software is employed.

## Conflicts of Interest

The authors declare no conflicts of interest.

## Supporting information




**Supporting File**: advs74285‐sup‐0001‐SuppMat.docx.

## Data Availability

The data that support the findings of this study are available from the corresponding author upon reasonable request.
