# Supplementary material for: Nanoimprinted DMD Electrodes Enabling Bidirectional Viewing OLEDs With Quasi Lambertian Emission
Source: Adv Sci (Weinh). 2026 Mar 2;13(22):e18921. doi: 10.1002/advs.202518921 (PMC13088353; doi:10.1002/advs.202518921)
Supplement: Supplementary file 1 — Supporting File: advs74285‐sup‐0001‐SuppMat.docx. [file ADVS-13-e18921-s001.docx]

**Supporting information for**

**Nano-imprinted DMD Electrodes enabling Bidirectional viewing OLEDs with Quasi Lambertian Emission**

Ningning Song, Ningning Liang*, Xinghao Guo, Ruixiang Chen, Xia Xin, Yiming Chen, Ruiqi Tian, Tianrui Zhai*

School of Physics and Optoelectronic Engineering, Beijing University of Technology, Beijing 100124, China

*Correspondence to: liangnn2020@bjut.edu.cn (N. N. L.), [trzhai@bjut.edu.cn](mailto:trzhai@bjut.edu.cn;) (T. R. Z.)

**Supplementary Figures**


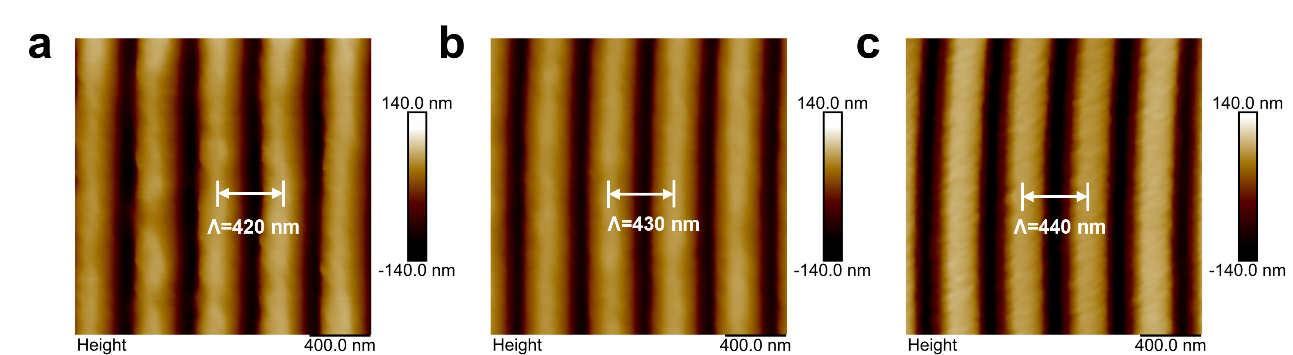


**Supplementary Figure S1.** Adjusting the dual-beam interference angle to obtain a periodically tunable grating structure. (a) 420 nm, (b) 430 nm, (c) 440 nm.


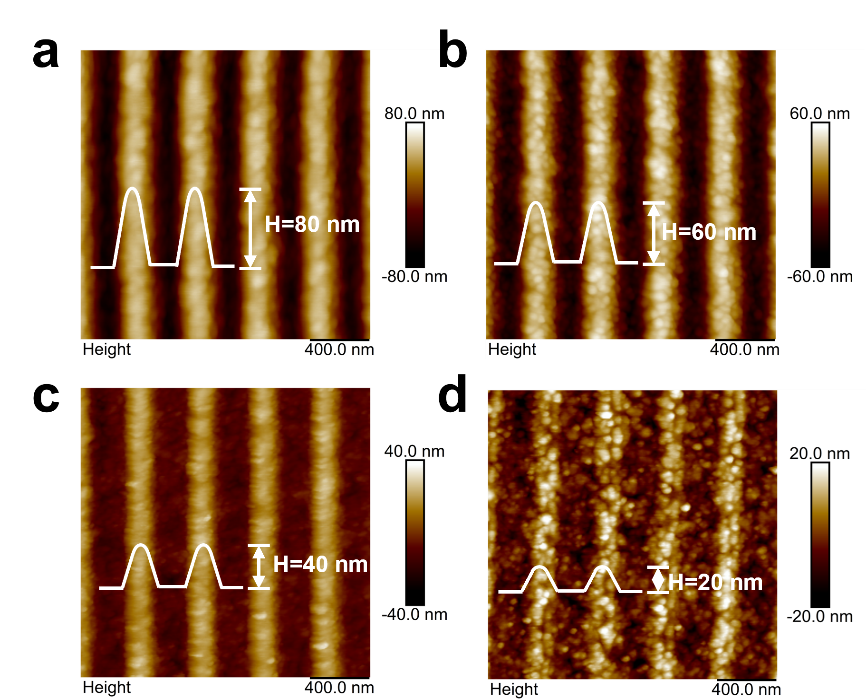


**Supplementary Figure S2.** Height-tunable grating structures obtained under different imprinting conditions. (a) 80 nm, (b) 60 nm, (c) 40 nm, (d) 20 nm.





**Supplementary Figure S3.** Comparison of refractive indices of materials. Refractive index and extinction coefficient of ETL, EML and HTL.


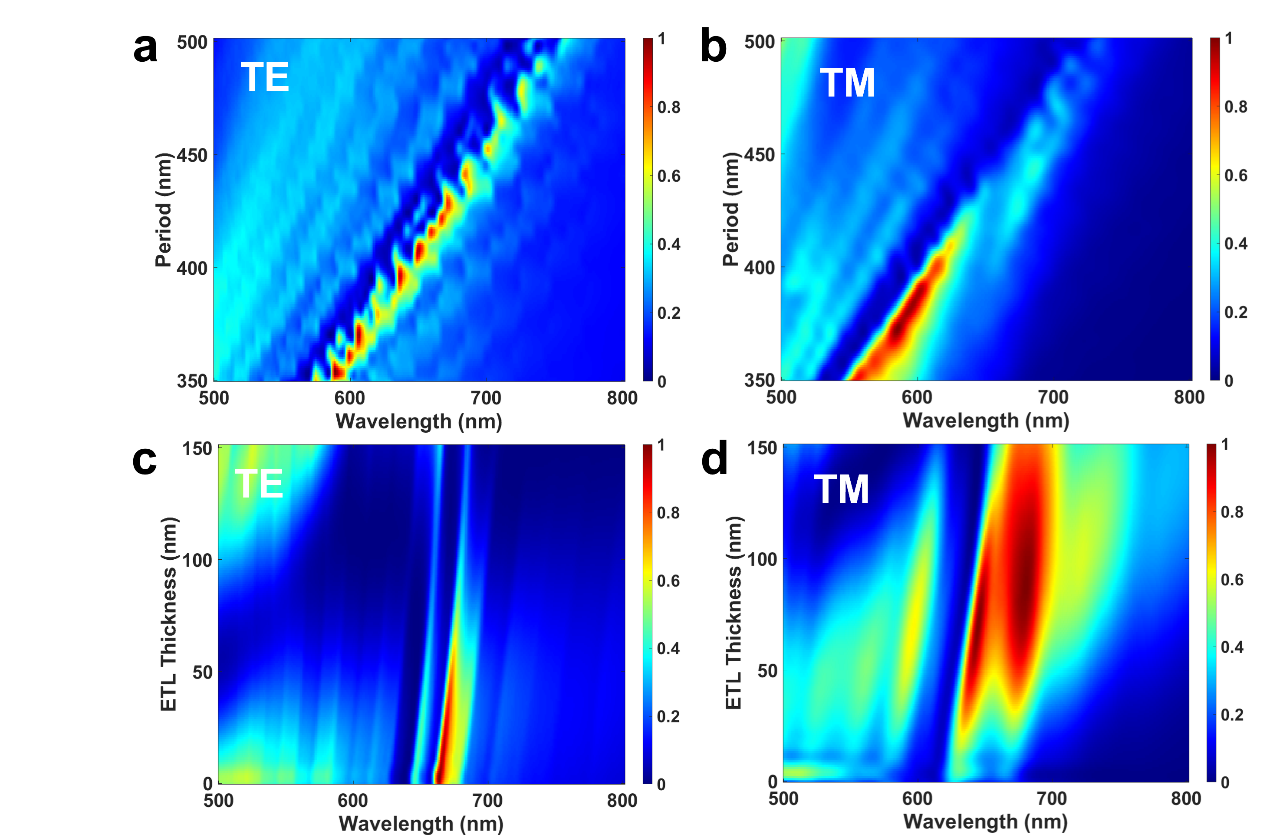


**Supplementary Figure S4.** Nanoimprinted OLED parameter simulation optimization. Far-field electric field distribution under different periods in (a) TE mode and (b) TM mode; far-field electric field distribution under different ETL thicknesses in (c) TE mode and (d) TM mode.


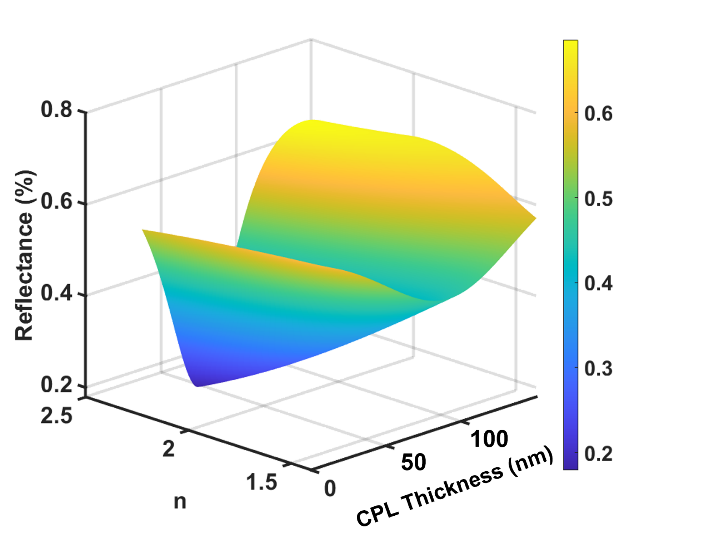


**Supplementary Figure S5.** Simulation optimization of the CPL layer. Reflectance as a function of CPL layer refractive index and thickness at a wavelength of 664 nm.


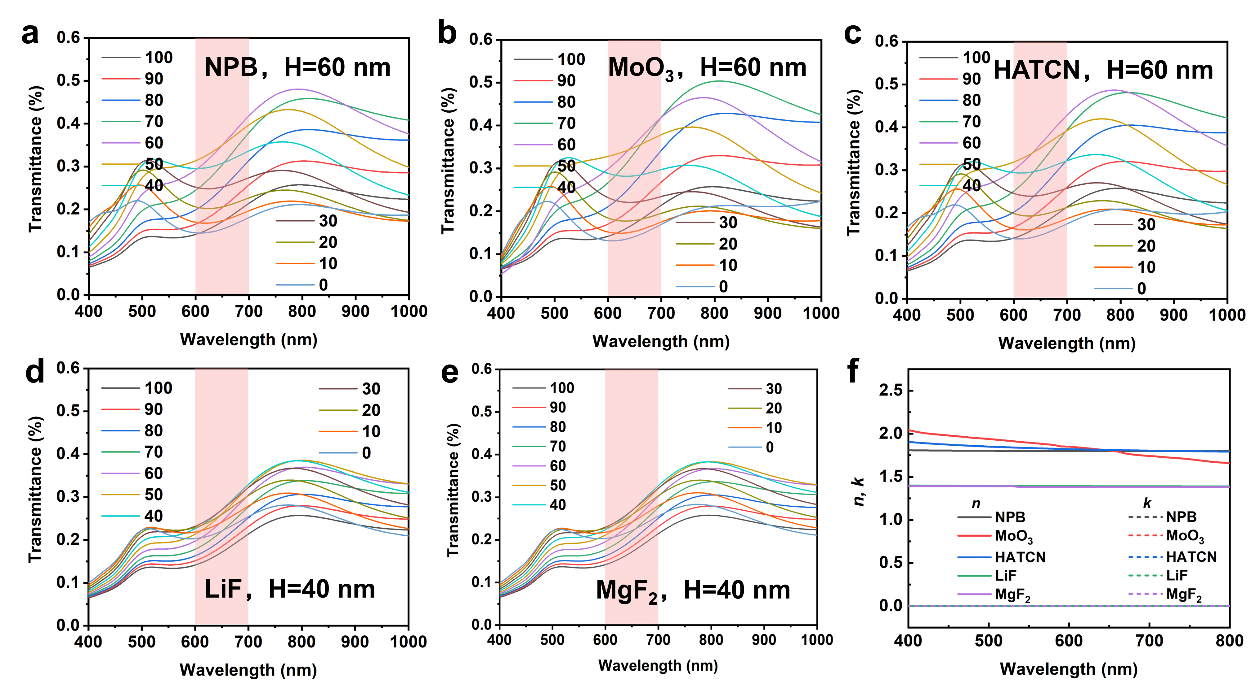
 **Supplementary Figure S6.** Device transmittance for different materials and thicknesses. (a) NPB, (b) MoO_3_, (c) HATCN, (d) LiF, (e) MgF_2_, and (f) refractive index.





**Supplementary Figure S7.** Reflectance of the CPL layer. p-mode Reflection at the upward direction as a function of CPL thickness and incident angle.


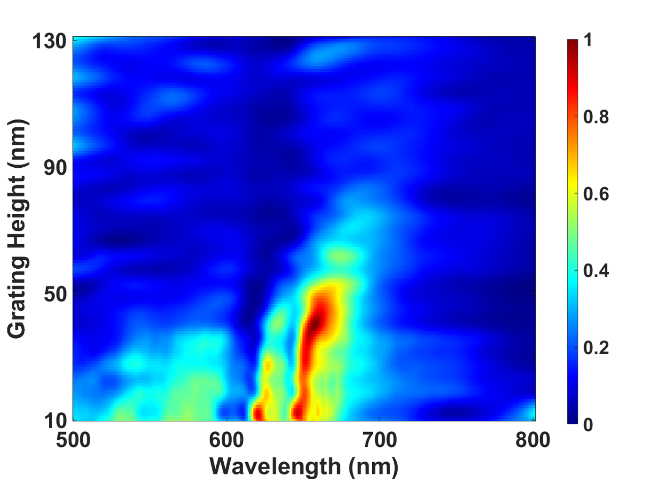


**Supplementary Figure S8.** Nanoimprinted OLED parameter simulation optimization. Far-field electric field distribution under different grating heights.


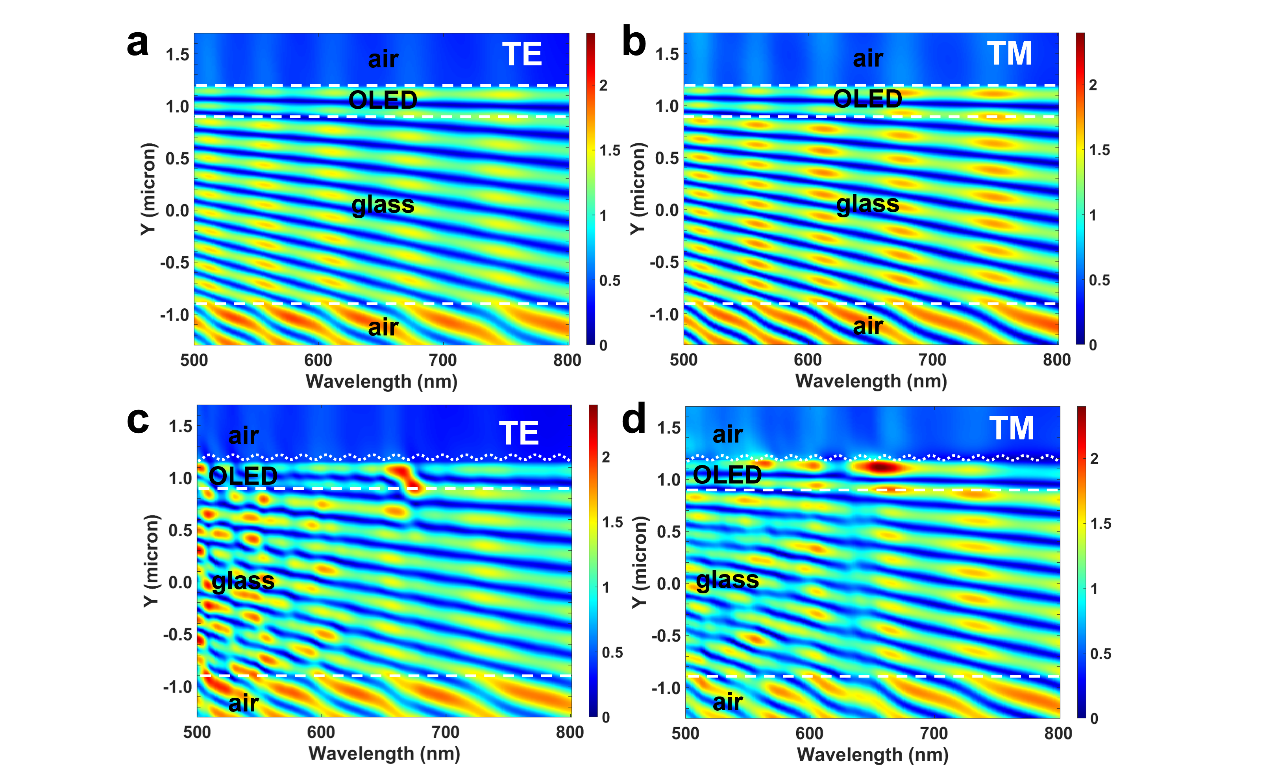
 **Supplementary Figure S9.** Internal electric field distribution under different polarization. Electric field distribution of planar OLEDs when incident plane light is in (a) TE mode and (b) TM mode; electric field distribution of nanoimprint OLEDs in (c) TE mode and (d) TM mode.


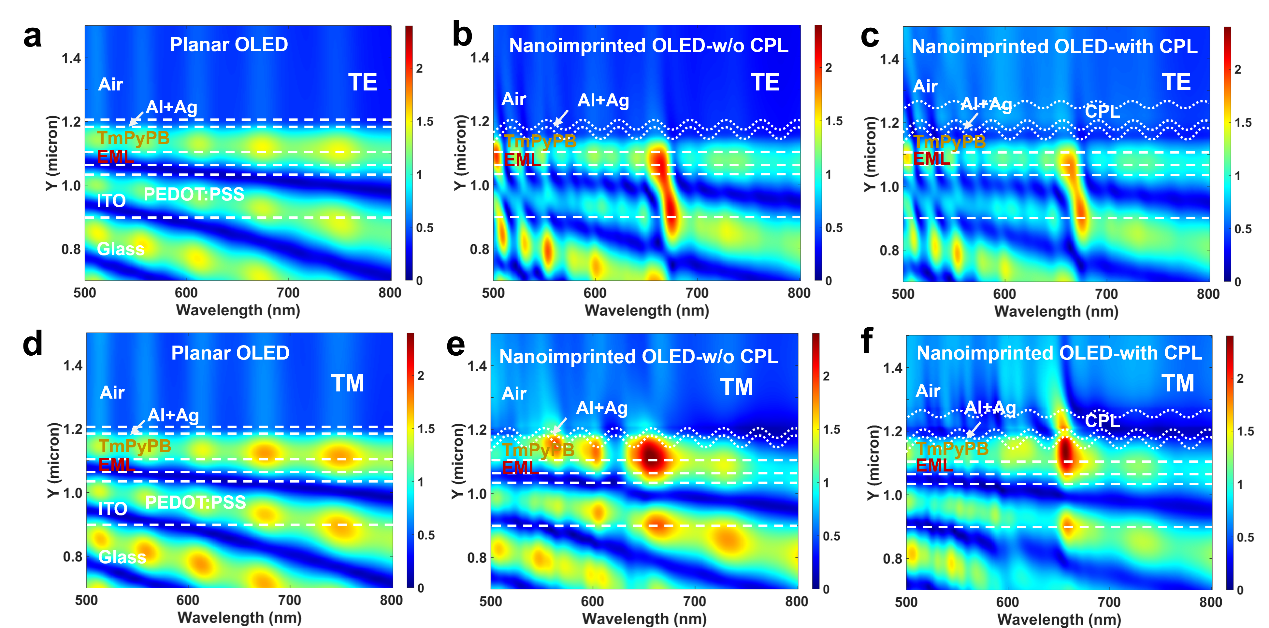


**Supplementary Figure S10.** Comparison of electric field distributions within the devices. (a) Planar OLED, (b) Nanoimprinted OLED-w/o CPL, (c) Nanoimprinted OLED-with CPL electric field distributions in TE mode; (d) Planar OLED, (e) Nanoimprinted OLED-w/o CPL, (f) Nanoimprinted OLED-with CPL electric field distributions in TM mode.


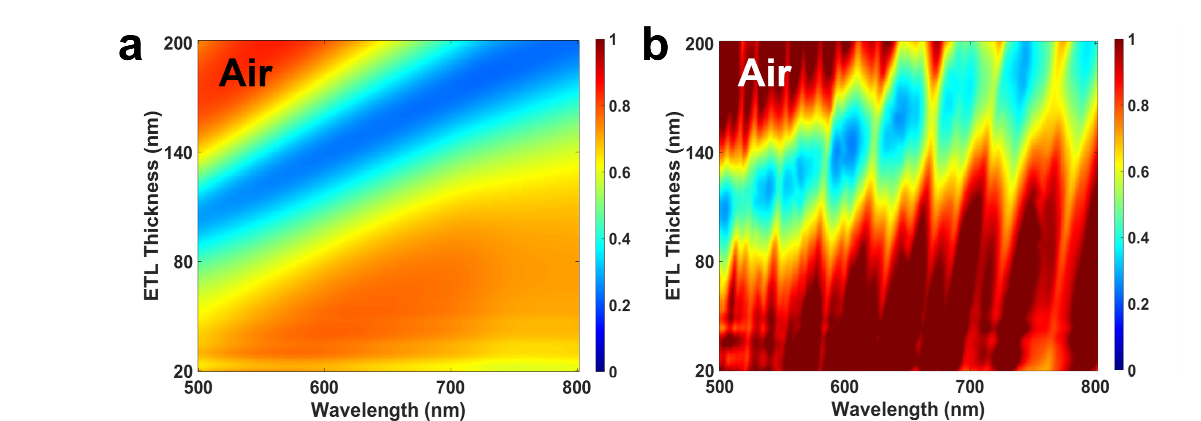


**Supplementary Figure S11.** Comparison of light extraction efficiency. Air mode distribution for (a) planar and (b) nanoimprinted OLEDs.


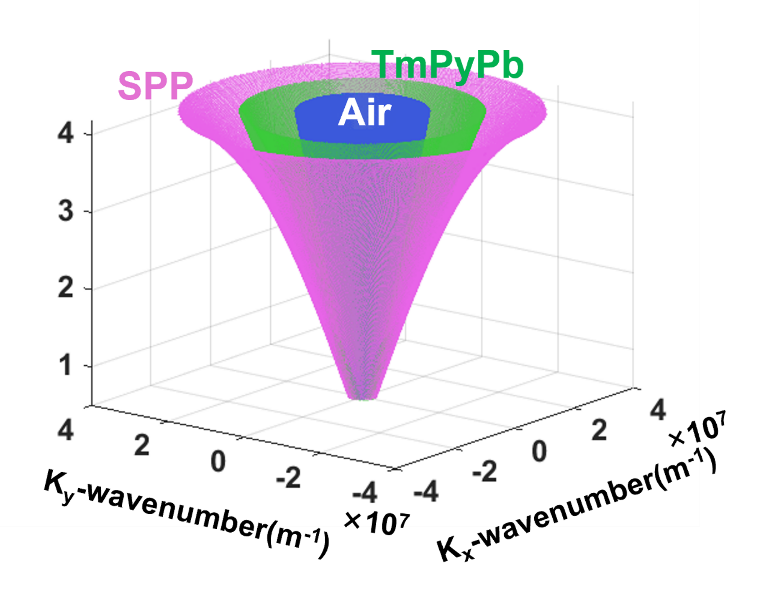


**Supplementary Figure S12.** Schematic diagram of photon momentum. Simplified three-dimensional photonic momentum distribution diagram for air, GaN, and SPP modes in a planar structure.


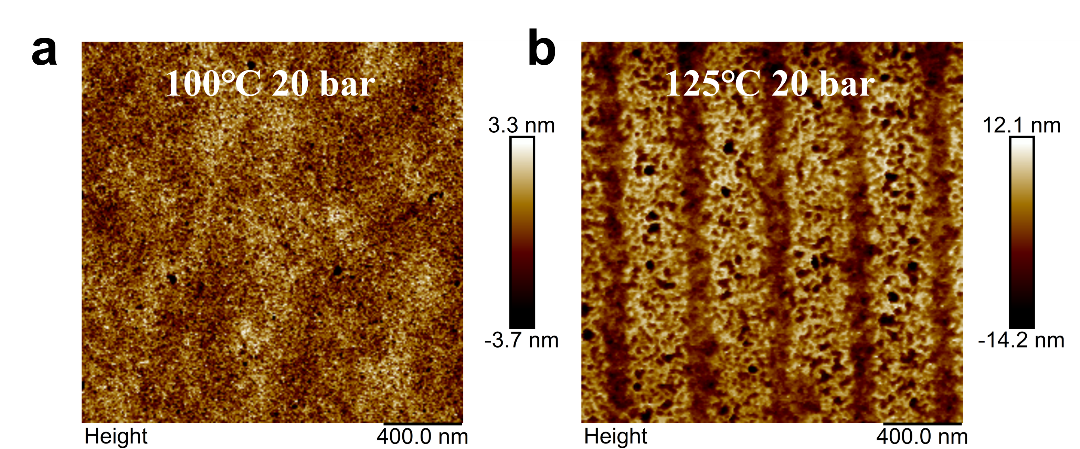


**Supplementary Figure S13.** The AFM images of the EML layer under different imprinting conditions.


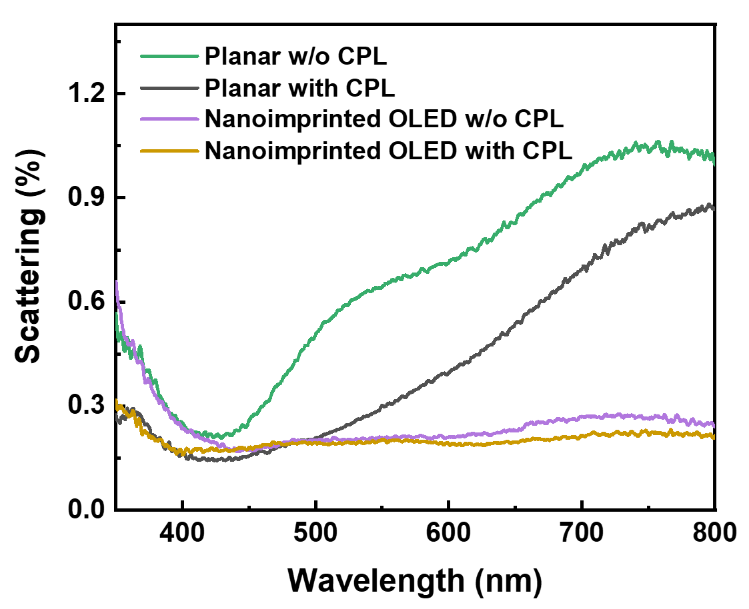


**Supplementary Figure S14.** Comparison of optical characteristics of devices. Scattering patterns of planar and nanoimprinted OLED devices with and without a CPL layer at different wavelengths.


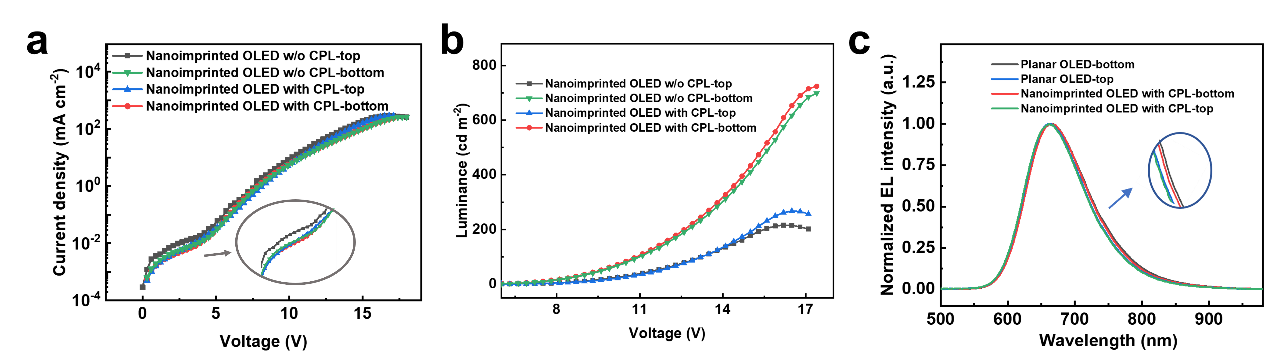


**Supplementary Figure S15.** Device performance comparison. (a) Current density and (b) brightness as a function of voltage for planar and nanoimprinted OLED devices with and without a CPL layer, and (c) EL emission peaks of planar and nanoimprinted OLED devices.


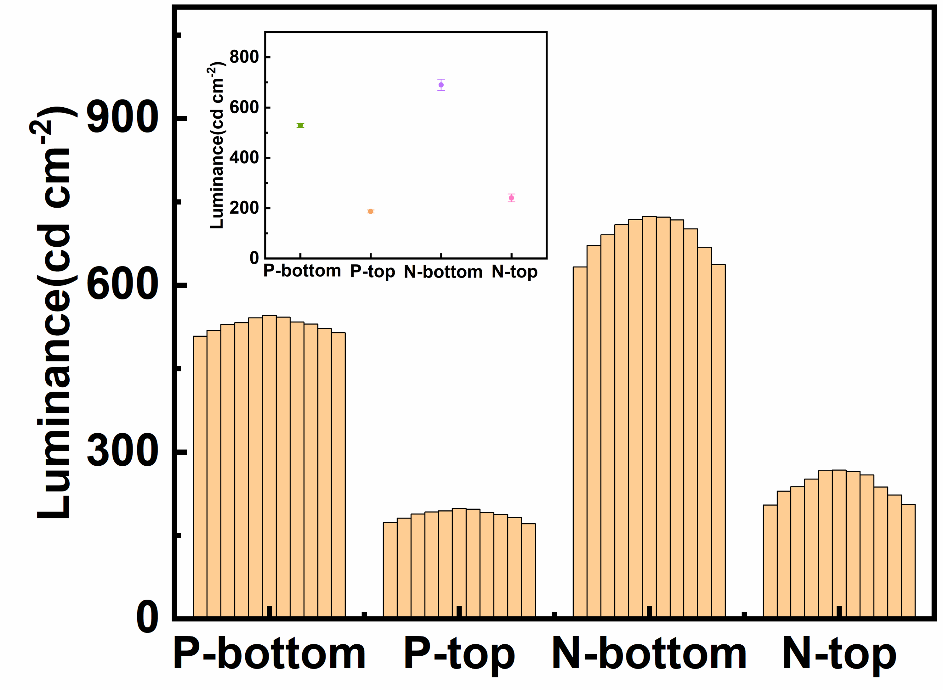


**Supplementary Figure S16.** Histograms demonstrate the Luminance of these four types of OLEDs. Here, P-bottom denotes Planar OLED-bottom, P-top refers to Planar OLED-top, N-bottom stands for Nanoimprinted with CPL OLED-bottom, and N-top indicates Nanoimprinted with CPL OLED-top.


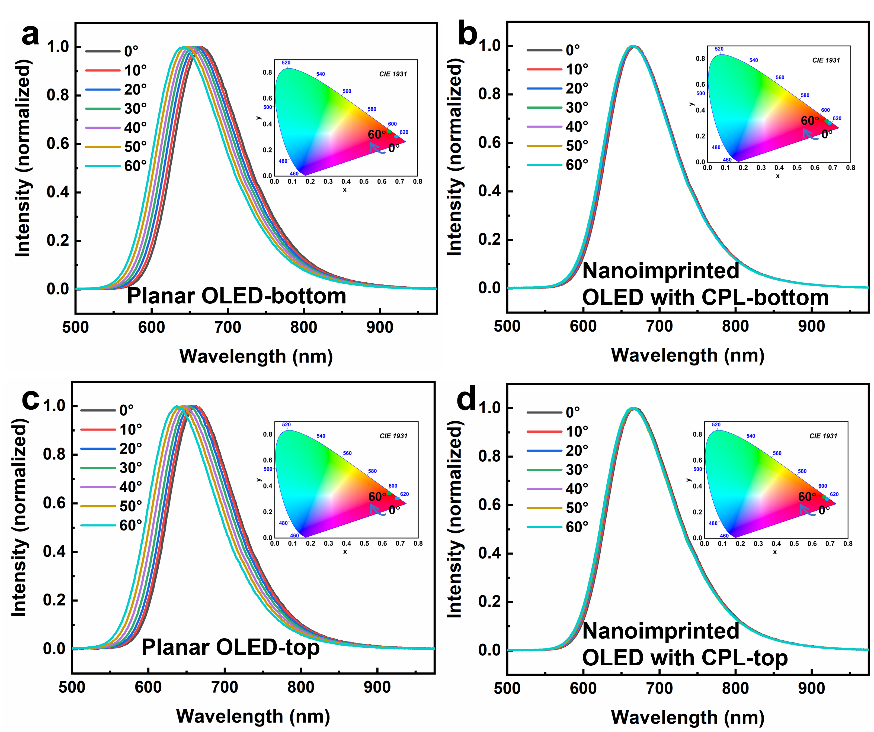


**Supplementary Figure S17.** EL spectra at viewing angles from 0° to 60°. Bottom-emission EL peak comparison of (a) Planar OLED and (b) Nanoimprinted OLED with CPL. Top-emission EL peak comparison of (c) Planar OLED and (d) Nanoimprinted OLED with CPL. The insets show the corresponding shifts in CIE coordinates.


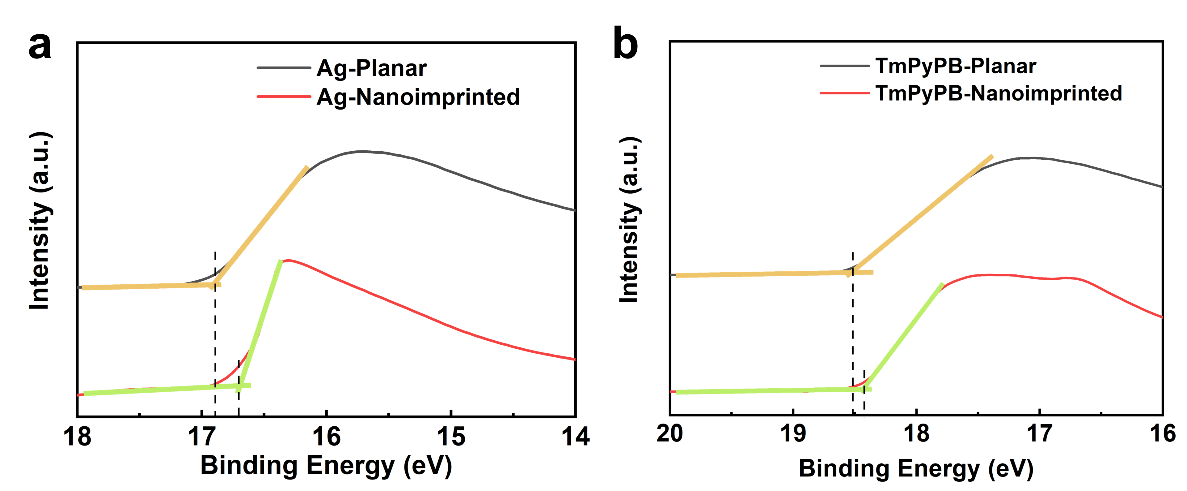


**Supplementary Figure S18.** UPS analysis of the work function for key interfaces. Secondary electron cutoff region for (a) the planar (top curve) and nanoimprinted (bottom curve) TmPyPB/LiF/Ag films, and (b)the planar (top curve) and nanoimprinted (bottom curve) TmPyPB surfaces.





**Supplementary Figure S19.** Comparison of device luminous stability. Brightness change trends measured at 5 mA injection current for top-emitting and bottom-emitting configurations of two unencapsulated OLEDs.

**Supplementary Notes**

**Supplementary Note 1-Expression of interference equation as a function of grating period with the target resonating wavelength and recording angle**

The fabrication of the period is given by the interference equation, which governs the coupling of wavelength and angle to adjust the period grating :

$$\begin{aligned} =\frac{}{2}\frac{1}{sin}\#\left（ 1 \right） \end{aligned}$$

The coated substrate was exposed to a 343 nm laser at a dose of approximately 70 mJ cm^-^², followed by development with a diluted developer, resulting in a photoresist with a grating structure with adjustable periodicity.

**Supplementary Note 2-The effect of refractive index and film thickness on transmittance and reflectance**

For continuous, uniform, isotropic parallel-plane thin films, the transmittance and reflectance of multilayer dielectric films can be calculated using the transmission matrix method (TMM). The propagation of light waves in a dielectric can be viewed as the superposition of forward-propagating electromagnetic waves (downward waves) and backward-propagating electromagnetic waves (upward waves). According to the electromagnetic field boundary conditions, the tangential components of the electric polarization E and magnetic polarization H at the interface between media are continuous. The interaction between each medium layer and the light wave is completely determined by its characteristic matrix. The magnitudes of the field vectors $E_{1}$, $H_{1}$, $E_{2}$, and $H_{2}$ on either side of the medium layer can be related by the characteristic matrix M:

$$\begin{aligned} \left[ \begin{matrix} E_{1} \\ H_{1} \end{matrix} \right]=M\left[ \begin{matrix} E_{2} \\ H_{2} \end{matrix} \right]\#\left（ 2 \right） \end{aligned}$$

In the formula, the transfer matrix M_j_ of the *j*th layer medium can be written as:

$$\begin{aligned} M_{j}=\left[ \begin{matrix} cos\delta_{j} & -\frac{i}{\eta_{j}}sin\delta_{j} \\ -i\eta_{j}sin\delta_{j} & cos\delta_{j} \end{matrix} \right]\#\left( 3 \right) \end{aligned}$$

$$\begin{aligned} \delta_{j}=\frac{2\pi}{\lambda}n_{j}d_{j}cos\theta_{j}\#\left（ 4 \right） \end{aligned}$$

$$\begin{aligned} \left\{ \begin{aligned} n_{j}=\frac{\sqrt{\varepsilon_{j}}}{\sqrt{\mu_{j}}}cos\theta_{j} （\mathrm{TE}） \\ n_{j}=\frac{\sqrt{\mu_{j}}}{\sqrt{\varepsilon_{j}}}cos\theta_{j} （\mathrm{TM}） \end{aligned} \right.\#\left( 5 \right) \end{aligned}$$

In this context, $\delta_{j}$denotes the phase shift of the light wave as it passes through the *j*th layer of medium, $n_{j}$ and $d_{j}$ represent the refractive index and thickness of the jth layer of medium, respectively, $\lambda$ is the wavelength of the incident light wave, and $\theta_{j}$is the angle between the propagation direction of the light wave and the normal direction of the interface. This represents the effective optical admittance of the medium. $\varepsilon_{j}$ and $\mu_{j}$ denote the relative permittivity and relative permeability of the *j*th layer of medium, respectively.

By applying the same principle layer by layer, the overall transmission matrix formula for a multilayer film structure can be derived:

$$\begin{aligned} \left[ \begin{matrix} E_{1} \\ H_{1} \end{matrix} \right]=M_{1}M_{2}\cdots M_{N}\left[ \begin{matrix} E_{N+1} \\ H_{N+1} \end{matrix} \right]=\prod_{j=1}^{N} M_{j}\left[ \begin{matrix} E_{N+1} \\ H_{N+1} \end{matrix} \right]=\left[ \begin{matrix} A & B \\ C & D \end{matrix} \right]\left[ \begin{matrix} E_{N+1} \\ H_{N+1} \end{matrix} \right]\#\left( 6 \right) \end{aligned}$$

By substituting the wavelength of the incident light wave, the incident angle, and the parameters of each layer of medium into the transmission matrix formula, we can obtain $E_{1}$, $H_{1}$, $E_{N+1}$, and $H_{N+1}$. The reflectance and transmittance can then be calculated using the following equations:

$$\begin{aligned} \left\{ \begin{aligned} &R=r\cdot r^{*}=\left| \frac{A\eta_{0}+B\eta_{0}\eta_{N+1}-C-D\eta_{N+1}}{A\eta_{0}+B\eta_{0}\eta_{N+1}+C+D\eta_{N+1}} \right|^{2} \\ &T=t\cdot t^{*}=\left| \frac{2\eta_{0}}{A\eta_{0}+B\eta_{0}\eta_{N+1}+C+D\eta_{N+1}} \right|^{2} \end{aligned} \right.\#\left（ 7 \right） \end{aligned}$$

**Supplementary Note 3-** **The effect of nanoimprinting on haze and its calculation method**

To quantitatively analyze the diffraction effect of nanoimprinting on devices, haze data was used for comparative analysis. The haze formula is as follows:$\begin{aligned} Haze=\frac{T_{diffuse}}{T_{total}}\times100\#\left（ 8 \right） \end{aligned}$

$T_{total}$represents the total transmittance measured using an integrating sphere, which captures light transmitted at all angles. $T_{diffuse}$ measures scattered light while excluding direct transmission ($T_{direct}$). Haze is therefore defined as the ratio of $T_{diffuse}$ to $T_{total}$.
